# What Contributes to the Activeness of Ethnic Minority Patients with Chronic Illnesses Seeking Allied Health Services? A Cross-Sectional Study in Rural Western China

**DOI:** 10.3390/ijerph120911579

**Published:** 2015-09-15

**Authors:** Shangfeng Tang, Dong Dong, Lu Ji, Hang Fu, Zhanchun Feng, Ghose Bishwajit, Zhifei He, Hui Ming, Qian Fu, Yue Xian

**Affiliations:** 1 School of Medicine and Health Management, Tongji Medical College, Huazhong University of Science and Technology, Wuhan 430030, China; 2 David C. Lam Institute for East-West Studies, Hong Kong Baptist University, Hong Kong, China; 3 Cancer Center, Sun Yat-sen University, Guangdong 510060, China

**Keywords:** activeness, allied health services, insurance, ethnic minorities, western China, rural areas

## Abstract

Actively seeking health services lies at the core of effective models of chronic disease self-management and contributes to promoting the utilization of allied health services (AHS). However, the use of AHS by ethnic minority Chinese, especially the elderly living in rural areas, has not received much attention. This study, therefore, aims to explore the association between personal characteristics and the activeness of ethnic minority patients with chronic diseases in rural areas of western China seeking AHS. A cross-sectional study was conducted to collect data on the socio-demographic and economic characteristics, health knowledge level and health communication channels of the sampled patients. A logistic regression model was used to examine the association of these predictors with the activeness of the surveyed patients in seeking AHS. A total of 1078 ethnic minorities over 45 years old who had chronic conditions were randomly selected from three western provinces in China and were interviewed in 2014. It is found that the New Cooperative Medical Scheme (NCMS) is the most salient predictor affecting the activeness of Chinese ethnic minorities in seeking AHS. The probability is 8.51 times greater for those insured with NCMS to actively seek AHS than those without (95% Confidence Interval (CI) 4.76–15.21; *p* < 0.001). Moreover, participants between 60 and 70 years old and those who have five to six household members are more likely to seek AHS compared with other social groups (Odds Ratio (OR) = 1.64, 95% CI 1.28–2.97, *p* = 0.007; OR = 1.95, 95% CI 1.15–2.36, *p* = 0.002). However, the activeness of patients seeking AHS is lower for those who have better household economic conditions. Besides socio-demographic predictors, the Chinese ethnic minorities’ activeness in seeking AHS is clearly associated with the communication channels used for receiving health information, which include direct communication with doctors (OR = 5.18, 95% CI 3.58–7.50, *p* < 0.001) and dissemination of elementary public health knowledge posted on bulletin boards (OR = 2.30, 95% CI 1.61–3.27, *p* < 0.001) and traditional mass media (OR = 1.74, 95% CI 1.22–2.48, *p* = 0.002). First, the government should further improve the coverage of NCMS to households suffering from chronic diseases and satisfy the requirements of social groups at different income levels and various ages in their health care to improve their activeness in AHS utilization. Second, doctors’ advice, bulletin boards and traditional media are common health communication channels for those seeking AHS and thus should be continuously employed in rural western China. Third, specified healthcare services should be designed to meet the needs of different patient segmentations.

## 1. Introduction

Chronic diseases are the leading cause of death in the world [1,2], and they are one of the major reasons individuals seek health care services spontaneously [3,4]. In China, the prevalence of chronic diseases has risen rapidly in the last decade. This increase has been followed by a worldwide trend in the consideration of the growing burden of non-communicable diseases as a serious public health concern [5]. As recent studies have shown, the burden of chronic diseases can increase considerably as the population ages [6,7,8]. In the United States, chronic diseases account for 75% of total healthcare expenditures [9]. Few governments in the underdeveloped world, however, can afford to shoulder the burden of expenses for treating chronic diseases [10]. The World Health Organization, among many other health organizations, has proposed a stepwise framework for effective intervention and prevention of chronic diseases [11].One of the most important key steps of the framework is to assess the current risk factors prevalent among the population of interest. Only by identifying the risk factors can further action be taken to solve the existing problems.

Previous research has shown that patients’ quality of life improves with increased utilization of health care [12]. The efficiency of patients’ utilization of allied health services (AHS) has been demonstrated to be crucial to enhancing their health status and is the core of effective models of chronic disease self-management [3]. Empirical evidence also shows that the more active and involved patients are in disease intervention, the more likely they will receive timely AHS and meet their health care needs [13]. In China, AHS, provided by primary health facilities, is a part of basic public health services. It mainly includes chronic disease examination, blood pressure and glucose monitoring, medication and healthy lifestyle and dietetics instructions [14], all of which are considered vital and effective control pathways for patients with chronic diseases [15]. Furthermore, there is a difference in China with other countries in allied health services, because they do not cover physiotherapy, occupational therapy, and speech therapy [16,17]. Given the importance of AHS to chronic disease self-management and the obvious link between patients’ activeness in seeking AHS and their health status, this study, therefore, investigates the factors contributing to the level of activeness with which chronic patients seek AHS, which is a topic that has not been sufficiently explored.

China is a developing country with 1.34 billion people consisting of 56 ethnic groups. The Han ethnicity constitutes the majority of the Chinese population. While the remaining 55 ethnic minority groups as a whole only make up 8.49% of China’s population, they still consist of 113.8 million residents [18]. Due to such ethnic diversity, promoting national unity and social harmony among different ethnic groups, particularly those in western China, is one of the main goals of the Chinese government. Therefore, public policies related to ethnic minorities in contemporary China must be investigated against specific political environments and socioeconomic conditions [19]. It is generally agreed that the rural region of western China is characterized by low economic status [20] and under-resourced health facilities [21]. Due to their distinctive historical and cultural backgrounds, ethnic minorities from those areas perhaps suffer even more than minorities from other areas. Although the government aims to offer preferential health policies in many under-resourced western villages and expects to generate significant changes in such areas, it is still unclear what the most important internal and external factors are that may affect the use of health services designed to benefit rural populations, including the ethnic minority Chinese farmers or herders. This research topic has recently attracted much interest among scholars [22,23]. Our study is no exception. As part of a comprehensive health intervention research project on residents in the rural areas of western China, this study was supported by the China Medical Board and the National Natural Science Foundation of China and was carried out between January 2011 and December 2014.

## 2. Methods

### 2.1. Study Design

As previous studies have estimated, a few types of chronic diseases—cardiovascular diseases, cancer, chronic respiratory diseases and diabetes—account for around 80% of chronic disease mortality in developing countries [6]. Among them, hypertension and diabetes are closely associated with high usage of healthcare services and resources [24,25,26,27]. Therefore, the main subjects of this study are people affected by these two chronic diseases who are ethnic minorities from rural western China (mainly Zhuang, Hui, Uygur and Mongolian ethnicities).

A cross-sectional survey was conducted in April 2014 using stratified multiple stage sampling. A five-step sampling procedure was employed. First, all of the 12 provinces/autonomous regions in western China were randomly divided into three parts, and one province was randomly selected from each part. Qinghai, Xinjiang and Inner Mongolia were chosen as our study sites. Second, all counties from the three provinces were divided into two groups according to their regional economic status, and one county was randomly selected from each group in rural regions (*i.e.*, 3 × 2 = 6 counties). Third, all townships in each of the sampled counties were divided into three groups, again based on their economic status, and one township was randomly selected from each group (*i.e.*, 3 × 2 × 3 = 18 townships). Fourth, all villages in each sampled township were grouped into three subgroups according to their distance to the township hospital, and one village was randomly selected from each subgroup (*i.e.*, 3 × 2 × 3 × 3 = 54 villages). Finally, 20 ethnic minorities with chronic conditions (*i.e.*, hypertension or/and diabetes) who had registered for resident health records were randomly selected from each of the 54 villages. A total of 1078 ethnic minority patients aged over 45 completed the survey. The response rate was 99.8% (1078/1080).

Ethical approval was obtained from the Ethics Committee of Tongji Medical College, Huazhong University of Science and Technology (IORG No: IORG0003571). The protocol, informed consent document and survey questionnaires were reviewed and approved. All participants were interviewed in person by investigators. Written consents were obtained from the participants at the beginning of the interview. Questions and response choices were read and explained when necessary.

### 2.2. Methods

The dependent variable was a binary variable indicating the activeness with which patients sought AHS. “Activeness” was measured by the actual use of AHS by an individual patient. For example, if the patients used AHS through a door-to-door service provided by doctors (from village clinics or township hospitals) or they only went to village clinics or a township hospital after receiving a reminder from doctor’s phone calls, we considered the patients “passive ” in AHS utilization and coded them as “no” (or the “passive group” hereafter). However, if the patients went to village clinics or township hospitals for AHS voluntarily and without any reminder, we considered them as “active” in seeking AHS and coded them as “yes” (or “active group” hereafter).

The accessibility of health-care services, such as NCMS coverage (that is, whether covered by the government-issued New Cooperative Medical Scheme), was obviously crucial to AHS usage and thus was treated as an independent variable. Socio-demographic characteristics [19,22,23], economic status [3], health knowledge level and health communication channels [15] have been shown to be important factors associated with chronic patients’ health behaviors. Hence, they were also included in the analysis.

The sixteen independent variables were: the accessibility of health-care services including (1) NCMS coverage, (2) distance and (3) time to reach the nearest health institution; socio-demographic and economic characteristics that included (4) age, (5) gender, (6) educational level, (7) occupation, (8) number of people in the household, (9) duration of illness and (10) annual disposable household income level; (11) health knowledge level, which was measured based on nine closed-ended questions related to chronic diseases; and health communication channels including (12) traditional media (that is, newspaper, radio or television), (13) new media (that is, the Internet ), (14) doctors, (15) bulletin boards and (16) family and friends.

### 2.3. Statistical Analysis

The sixteen independent variables were first summarized and explored using descriptive statistics. Chi-square tests were then employed to compare the covariates of interest between the active and passive groups. Only the variables with statistically significant differences between the two groups were subsequently included in the logistic regression analysis model using the Wald statistic with forward stepwise selection. In this study, the cross-sectional data were doubly entered into the EpiData3.1 database and statistics program (Atlanta, GA, USA) and data entry screens were used to revise incorrect entries (*i.e.*, input and logic errors). Statistical analysis was implemented using SPSS 13.0 (SPSS Inc., USA). All promulgated *p*-values were two-sided, and the statistical significance test level was set at 0.05.

## 3. Results

### 3.1. Sample Characteristics

As shown in Table 1, more than half of survey participants are elderly people more than 60 years old (66.0%). Females and males are almost evenly distributed in the sample (51.3% and 48.7%, respectively). Most participants are farmers and herders (85.7%). More than 60% of participants have less than six years of education (61.2%), and thus a similar proportion of them show a health knowledge level lower than the average population (62.7%). This finding coincides with a previous study that identified a relatively high prevalence of low health literacy among ethnic minorities [28].

In accordance with the Chinese government’s efforts to prevent rural residents from being impoverished by medical expenses, more than 93% of participants are covered by NCMS. Also, most villagers live quite close to a particular health facility (such as village clinics or township hospitals), within a one-kilometer radius or less than a 20-min walking or driving distance (depending on the usual transportation method that the participant uses to get to the health facility). Since ethnic minorities in China are exempt from the one-child policy, more than half of participants have more than five members in their households, which is larger than the average household size (Number = 3.02 in 2012) in China [29]. However, the annual disposable household income of the ethnic minority participants on average seemed quite low. More than 80% of the families were living with an annual income below 30,000 RMB (approximately 4800 US dollars) per year, which is consistent with the majority’s self-evaluation of their own income level, as low to middle.

In terms of the communication channels they used to retrieve health-related information, the most popular channels were doctors and family/friends (78.7% and 64.7%, respectively). The new media coverage ratio is the lowest among the channels for ethnic minorities in rural western China, only 12.8% of them use it to receive health-related information (see Table 1).

**Table 1:** Socio-demographic characteristics and economic status of ethnic minority patients with chronic diseases in rural western China, their accessibility of healthcare services, health knowledge level and health communication channels.

Characteristics	Frequency (n = 1078)	Percentage (%)
**Socio-demographic characteristics**		
**Age**		
45–59	367	34.0
60–69	384	35.6
>70	327	30.3
**Gender**		
Male	525	48.7
Female	553	51.3
**Education level**		
Less than 6 years study	661	61.3
6–9 years study	287	26.6
Over 9 years study	130	12.1
**Occupation**		
Farmer/herders	924	85.7
Self-employed or migrant worker	45	4.2
Retired or non-work	109	10.1
**Number of people in household**		
<5	358	33.2
5–6	591	54.8
>6	129	12.0
**Economic status**		
**Annual disposable household income**		
<RMB10,000	273	25.3
RMB10,000–RMB29,999	538	49.9
RMB30,000 and above	267	24.8
**Self-assessment of household income level**		
Low	256	23.8
Middle	581	53.9
High	241	22.4
**Accessibility of healthcare services**		
**New cooperative medical insurance**		
Yes	1003	93.0
No	75	7.0
**Distance to the nearest health facilities (Km)**		
<1	951	88.2
1–2	71	6.6
>2	56	5.2
**Time to reach the nearest health facilities (Min)**		
<10	659	61.1
10–19	323	30.0
>20	96	8.9
**Accessibility of healthcare services**		
**Health knowledge level**		
Below average	676	62.7
Above average	402	37.3
**Health Communication Channels ^1^**		
Traditional media	501	18.5
New media	138	5.1
Doctors	848	31.3
Bulletin boards	527	19.4
Family and friends	697	25.7

Note: ^1^ Respondents could choose more than one health communication channels and thus the sum of frequency here is larger than 1078.

### 3.2. The Activeness of Ethnic Minorities with Chronic Diseases in Seeking AHS

Among the 1078 rural Chinese ethnic minorities, 75.5% of them reported that they actively utilize AHS. Table 2 shows the comparison between the active and passive groups in terms of their socio-demographic (*i.e.*, age, gender, occupation and household size) and economic characteristics, accessibility to health-care services, health knowledge level and the communication channels they use to receive health information related to chronic diseases. Significant differences are found between the active and the passive AHS seekers on many of these variables.

The active AHS seekers seem more likely to be rural Chinese ethnic minorities who are farmers or herders between 60 and 69 years old, with five to six household members and with low to middle household income (both annual disposable and self-assessment). They are more likely to be covered by NCMS and to obtain health information from all types of communication channels, whether it is directly from their doctors or mass mediated by traditional media or the village bulletin boards. However, the activeness in seeking AHS does not seem to be associated with the survey participants’ gender, their educational level and related health knowledge level and the distance and time they need to visit the nearest health facilities. The new media or the villagers’ family and friends do not seem to influence their decision in using AHS either.

### 3.3. Predictors Affecting the Activeness of Ethnic Minorities with Chronic Diseases in Seeking AHS

After discovering that gender, educational level and other factors are not significantly associated with activeness in seeking AHS, these factors were excluded from the original model. A binary logistic regression analysis was then implemented to examine the potential predictors of the activeness of ethnic minority chronic patients in seeking AHS in rural western China. Eight predicting variables, including age, NCMS coverage, number of people in households, household income and the three health communication channels (traditional media, bulletin boards and doctors), were finally retained in the binary logistic regression model.

**Table 2:** Correlations between the activeness of rural ethnic minority Chinese with chronic diseases in seeking AHS and their socio-demographic characteristics, economic status, health knowledge level, accessibility of health-care services and their use of health communication channels.

Associated Correlates	Passive Group Seeking AHS * (n = 264)			Active Group Seeking AHS * (n = 814)		χ^2^	*p*
	N	%		N	%		
**Socio-demographic characteristics**							
**Age**						42.61	**<0.001**
45–59	98	37.12		269	33.05		
60–69	53	20.08		331	40.67		
>70	113	42.80		214	26.29		
**Gender**						0.24	0.671
Male	132	50.00		393	48.28		
Female	132	50.00		421	51.72		
**Education level**						2.56	0.278
Less than 6 years study	167	63.26		494	60.69		
6–9 years study	61	23.11		226	27.76		
Over 9 years study	36	13.64		94	11.55		
**Occupation**						13.77	**0.001**
Farmer	208	78.79		716	87.96		
Self-employed or migrant worker	17	6.44		28	3.44		
Retired or non-work	39	14.77		70	8.60		
**Number of people in household**						15.58	**0.001**
<5	106	40.15		252	30.96		
5–6	118	44.70		473	58.11		
>6	40	15.15		89	10.93		
**Economic status**							
**Annul disposable household income**						20.59	**<0.001**
<RMB10,000	59	22.35		214	26.29		
RMB10,000–RMB29,999	112	42.42		426	52.33		
RMB30,000 and above	93	35.23		174	21.38		
**Self-assessment of household income level**						6.14	**0.047**
Low	50	18.94		206	25.31		
Middle	144	54.55		437	53.69		
High	70	26.51		171	21.00		
**Accessibility of healthcare services**							
**New cooperative medical insurance**						98.39	**<0.001**
Yes	210	79.55		793	97.42		
No	54	20.45		21	2.58		
**Distance to the nearest health facilities (Km)**						1.96	0.375
<1	239	90.53		712	87.47		
1–2	13	4.92		58	7.12		
>2	12	4.55		44	5.41		
**Accessibility of healthcare services**							
**Time required to the nearest health facilities (Min)**						1.89	0.391
<10	165	62.50		494	60.69		
10–19	81	30.68		242	29.73		
>20	18	6.82		78	9.58		
**Health knowledge level**						0.05	0.820
Below average	164	62.12		512	62.90		
Above average	100	37.88		302	37.10		
**Health Communication Channels**							
**Traditional media**						33.39	**<0.001**
Yes	82	31.06		419	51.47		
No	182	68.94		395	48.53		
**New media**						1.73	0.188
Yes	40	15.15		98	12.04		
No	224	84.85		716	87.96		
**Doctors**						65.11	**<0.001**
Yes	161	60.98		687	84.40		
No	103	39.02		127	15.60		
**Bulletin boards**						29.08	**<0.001**
Yes	91	34.47		436	53.56		
No	173	65.53		378	46.44		
**Family and friends**						0.02	0.964
Yes	171	64.77		526	64.62		
No	93	35.23		288	35.38		

Note: *AHS = Allied Health Services.

Among all significant predictors, the odds of rural Chinese ethnic minorities who are insured by NCMS to actively seek AHS was 8.51 times greater than those who are not insured (OR = 8.51, 95% Confidence Interval (CI) = 4.76–15.21; *p* < 0.001). Compared with low-income families (annual disposable household income lower than 10,000 RMB), when the household income status is higher, the activeness of patients with chronic diseases seeking AHS, however, seems to decrease. The rural ethnic minority Chinese aged between 60 and 70 are more likely to seek AHS than those aged below 60 (OR = 1.95, 95% CI 1.28–2.97, *p* = 0.002). However, among the older patients, the activeness in seeking AHS decreases sharply. Moreover, family size also seems to matter. The activeness of the rural patients living with five to six family members was 1.644 times greater than those with fewer than five members (OR = 1.64, 95% CI 1.14–2.36, *p* = 0.007). Whereas, there is no difference between them and those with over six members (OR = 0.90, 95% CI 1.15–2.36, *p* = 0.672). At last, as expected, whether one receives health information related to chronic diseases through traditional media, bulletin boards and doctors is highly related with ethnic minority patients actively seeking AHS (OR = 1.74, 95% CI 1.22–2.48, *p* < 0.001; OR = 2.30, 95% CI 1.61–3.27, *p* < 0.001 and OR = 5.18 , 95% CI 3.58–7.50, *p* < 0.001).

**Table 3:** Outcome of a logistic regression analysis model examining predictors correlated with the activeness of minorities with chronic disease seeking AHS.

Predictors	Reference	B	*p*	OR	95% CI	
					Lower	Upper
**New cooperative medical insurance**	No	2.14	**<0.001**	8.51	4.76	15.21
**Age**	45–59		**<0.001**			
60–70		0.67	**0.002**	1.95	1.28	2.97
>70		−0.50	**0.009**	0.61	0.42	0.89
**Number of people in household**	<5		**0.006**			
5–6		0.50	**0.007**	1.64	1.15	2.36
>6		−0.11	0.672	0.90	0.54	1.49
**Annual disposable household income**	< RMB10,000		**0.001**			
RMB10,000–RMB29,999		−0.18	0.427	0.83	0.53	1.31
RMB30,000 and above		−0.85	**0.001**	0.43	0.25	0.71
**Self-assessment of household income level**	Low		**0.018**			
Middle		−0.57	**0.009**	0.56	0.360	0.86
High		−0.69	**0.012**	0.50	0.29	0.86
**Traditional media**	No	0.55	**0.002**	1.74	1.22	2.48
**Doctors**	No	1.65	**<0.001**	5.18	3.58	7.50
**Bulletin boards**	No	0.83	**<0.001**	2.30	1.61	3.27
**Constants**		−2.06	**<0.001**	0.13		

## 4. Discussion

This study showed a number of factors that influence the activeness of Chinese ethnic minorities with chronic non-communicable diseases in AHS utilization. Different from previous studies, these predictors, however, do not include gender, educational level, health knowledge level [15,30], and distance and time to the nearest health facilities. In prior research, it was widely believed that people suffering from various chronic diseases are sensitive to utilizing AHS [31], especially the female patients who bear the burden of taking care of their families [32]. Furthermore, based on the model of KABP (Knowledge, Attitude, Belief, Practice), knowledge is the foundation of attitude. This means that the more health knowledge the patients have acquired, the higher the likelihood they would actively seek AHS. Surprisingly, no such correlation was found between health knowledge and activeness of patients seeking AHS. This is perhaps because of low educational level, and, hence, the health knowledge level among rural ethnic Chinese minority is too low to actually affect their decisions about seeking AHS.

The burden on society is aggravated with the aging of population, the same as for individuals with chronic diseases. A previous study found that the utilization of health care services appears to increase significantly with age [12]. However, our study found that “age” needs more in-depth examination. As we subdivided the rural ethnic minority Chinese patients into three groups—younger than 60, between 60 and 70 and older than 70—the results showed that those aged between 60 and 70 were the most active in seeking AHS. The activeness of those older than 70 was significantly lower than those who were younger. This can probably be explained by the fact that the disposable time available for health care greatly increases after patients are freed from their work and other social obligations at the age of 60, commonly known as the retirement age.

Despite the socio-demographic factors, the problem with rural Chinese ethnic minorities’ activeness in seeking AHS seems to be “economic” in nature. On the one hand, we found that the New Cooperative Medical Scheme (NCMS), designed to reduce the financial burden of disease among the rural population, is the most critical factor affecting their use of AHS. Established in 2003, NCMS covers more than 90% of the population in rural China and has substantially reduced the financial burden of healthcare for rural populations [33,34]. For chronic patients, two AHS items are covered by NCMS: medications and informal examinations (such as routine blood examination). In recent years, the NCMS reimbursement rate was raised to 50% for outpatient and 95% for inpatient treatment at township hospitals [34]. This elevated reimbursement level has attracted many ethnic minorities in rural areas, especially those from lower-income families who have suffered health disparities for economic reasons.

The results indicated that the NCMS program has the highest influence regarding healthcare seeking behavior among ethnic minorities. This coincides with our finding that patients with chronic illnesses who have low household incomes are more active in seeking AHS. There might be two additional reasons. First, the elderly from low-income families might be more interested in free-of-cost services. As former research reveals, the pandemic of chronic diseases affects the poor disproportionately [35]. The economic and social burden of diseases is much heavier for those who live in poverty than those with better economic conditions. In China, most of the AHS purchased by government is provided for free. Apart from monitoring and controlling blood pressure or glucose levels, AHS also reduces the financial burden of chronic diseases for the patients’ families, which seems most helpful for those who do not have sufficient economic resources for paid services. This may also, in turn, explain why NCMS coverage has such a significant impact on AHS utilization. Second, being free-of-cost in nature, AHS does not seem to be able to meet the more demanding healthcare needs of high-income social groups. It is generally believed that the attractiveness of AHS is not as significant for patients with chronic illnesses who come from high-income families as for those from low-income ones [36]. For patients from high-income families, price is less of a concern than the quality of healthcare services. Our study suggested that it is important to differentiate and pluralize healthcare services, including AHS, to motivate social groups with different income levels to actively participate in healthcare related programs.

Health communication is regarded as a core component in public health promotion and intervention in rural areas [15,37]. In rural China, the conduits used for communicating health information include traditional and new media, interpersonal networks, such as doctors, families and friends and bulletin boards in villages. Our study revealed that not all of these conduits work with equal efficiency. Only doctors, bulletin boards and traditional mass media were significantly associated with the activeness of the rural ethnic minorities in seeking AHS. However, this does not mean that we should ignore the importance of new media. As times change and the younger population ages, new media may become more available in rural areas and, thus, play more important roles in communicating health information. Previous studies have shown that the trust of patients in doctors is an important predictor of the patients’ health seeking behaviors [37]. Support from families and friends is not less important. However, in our study, only doctors—not families and friends—were found to be influential in the rural chronic patients’ AHS usage. Interestingly, bulletin boards were also found to be of considerable influence despite the low educational level of survey participants. This may further explain why their AHS use was not affected by families and friends but by doctors.

## 5. Limitations

There are several limitations of this study. First, the survey results only included patients with chronic hypertension and/or diabetes, which may have left other chronic diseases unstudied. Second, the data is based on patients’ self-reporting, which certainly is subjected to individual variances. Factors such as the distance and time required to reach the nearest health institution or self-assessment of household income level are quite subjective and hard to estimate. Hence, we have to assume that the assessment criteria are the same for every participant. Third, the health knowledge level is tested through closed-ended questions, which may have allowed patients to guess the correct answers. Finally, the measurement of the activeness of patients seeking AHS is only a one-item question that enquires about their use of AHS in health institutions.

## 6. Conclusions

Actively seeking healthcare services is critical for the health management of rural Chinese ethnic minorities with chronic diseases. It is, therefore, important to promote the allied health service utilization among this population. In this study, we intended to examine the associations between the activeness in seeking AHS by rural ethnic minority patients with chronic diseases and their socio-demographic characteristics, economic status, accessibility of healthcare services, health knowledge level and health communication channels. The study revealed that the NCMS coverage plays the most prominent role in encouraging patients to utilize AHS, and factors such as age, household income level and the number of household members were less strongly associated to care seeking behavior. In addition, doctors, bulletin boards and traditional media were found to be common health communication channels in western rural China. Based on the findings, we suggest that the government further improve the coverage and protection of NCMS to the rural population suffering from chronic diseases and provide more specified health-care services, including AHS, to meet the complex healthcare needs of different patient segmentations.
